# Village or Tribe? Expectations, Roles, and Responsibilities for Effective Fall Prevention Efforts

**DOI:** 10.3389/fpubh.2014.00163

**Published:** 2015-04-27

**Authors:** Tiffany E. Shubert

**Affiliations:** ^1^Division of Geriatric Medicine, Center for Aging and Health, University of North Carolina at Chapel Hill, Chapel Hill, NC, USA

**Keywords:** fall prevention, aging, evidence-based practice, physical therapy, systems-change, policy

*Village: A group of houses and associated buildings, larger than a hamlet and smaller than a town*.

*Tribe: A social division in a traditional society consisting of families or communities linked by social, economic, religious, or blood ties, with a common culture and dialect. Oxford Dictionary*.

Effective fall prevention efforts bridge the silos between clinical and community practice. A fall experienced by an older adult is rarely a straightforward event. Typically, falls are due to complex inter-related medical, behavioral, and environmental risk factors (1). For many older adults, medical risk factors such as medication reconciliation, treatment of atrial fibrillation, or physical therapy to address gait and balance impairments are primary in fall prevention (2). However, this is only the beginning of the fall prevention story.

Once medical risk factors are managed, the focus of risk management should transition to behavioral and environmental factors (3). This will ensure that the older adult has the ability to safely interact with their environment to prevent a future fall. One of the most robust interventions is strength and balance training to minimize fall risk (4). Two hours of strength and balance training done each week is the minimum dose required to effectively prevent a fall or fall-related injury (5).

To achieve this dose of exercise typically requires a behavior change (6). Established protocols to transition from a clinically supervised rehabilitation program to an evidence-based community program will support this behavior change. Once the initial clinical-community transition is complete, to further support behavior change, the older can be embedded into the continuum of the community. For example, the older adult could move from programs that target the more frail and deconditioned, like Stepping On (7), to those that target more robust individuals, like Tai Chi (8).

This proposed model supposes that infrastructure is in place to build a continuum of care where none exists. To achieve this model, stakeholders have called for multi-level, multi-component interventions, with the goal of engaging policy makers, healthcare providers, community providers, and older adults themselves. Many have compared these efforts to building a “village” of providers (9).

The concept of “village” is appealing, though may be inherently flawed. A village is a group of buildings that simply share the same physical location. These buildings are not necessarily inter-related, inter-dependent, or even connected by a common culture or value system. Besides being in the same physical location, there is no common commitment among members of a village.

This scenario of assumed but not confirmed alignment of priorities and goals often plays out in fall prevention. Many public health providers mistakenly assume that healthcare providers integrate fall risk screening and management into their practices. For example, an evidence-based fall prevention exercise program is offered in the community. An older adult is interested in attending the program, and must be cleared by their physician before participating. The older adults request a falls screen from their physician. The physician, however, does not understand her expected role in fall prevention. She has not been trained in fall screening. She assumes that if the patient is asked to be screened then she is at risk of falling, and is not going to be safe in the community program. This is not an atypical behavior; studies have shown that less than 30% of healthcare providers who interact with older adults screen for falls on a routine basis (10).

Physical therapists are also uncertain about their roles and responsibilities in the fall prevention continuum. For example, few physical therapists are aware of evidence-based programs that target populations at risk of falling (11). They also may not understand the role of State Fall Prevention Coalitions, or perceive them as partners in creating a continuum. In a survey of PTs interested in disseminating the Otago Exercise Program (OEP), the majority of PTs indicated that support of a program by State-Based Fall Prevention Coalitions was not a facilitator to program implementation (11).

A similar story exists from the public health perspective. State-Based Fall Coalitions identified working with healthcare providers to disseminate evidence-based fall prevention programs as a top priority (12). However, it is clear that a disconnect exists between the expectations and actions of healthcare providers by the Coalitions may be resulting in gaps in the continuum.

A final example is the complex and misunderstood role of older adults. Though almost all Fall Prevention Coalitions have the goal of education and public awareness, few, if any, actually have older adults as active members of their coalitions (12). Preliminary evidence from pilot studies supports a disturbing trend that even by educating healthcare providers and offering innovative programing, many older adults are likely to refuse when offered an intervention to minimize their risk for falling.

A “tribe” differs from a “village” in that there is a shared common culture and values. Everyone has a prescribed job to achieve the common goals. For effective multi-level fall prevention efforts to happen, we may want to shift the paradigm from assuming each stakeholder understands their roles to describing and motivating stakeholders to be part of a shared social movement.

What would this look like for future efforts? Current tribe building efforts have demonstrated success. For example, the Oregon State Department of Public Health (DPH) engaging the state chapter of the American Physical Therapy Association to educate physical therapists about the role of public health in fall prevention and providing partnership models. This partnership was designed to facilitate the implementation of the OEP. It was discovered that physical therapists were not familiar with the OEP. Once they were invited to engage with the program, and supported by the Oregon State DPH, OEP adoption and implementation rates increased. The Community Health Worker (CHW) Training described by St. John et al. is another example (13). The goal is to educate CHWs about their role in fall prevention, and in turn to help the CHWs educate their older adult clients. This will ensure that the CHW can contribute to the tribe by contributing to a knowledge base they can use to educate and engage with other healthcare providers who care for their clients (13).

Starting with a small group and crossing between disciplines, educating and engaging all key players, and building a common culture of fall prevention will be the key to creating an effective tribe. Every member, no matter how old or young, licensed professional or community provider, has a significant role to play, they just may not know it yet. As we move forward in dissemination and implementation of evidence-based fall prevention programs nationally, the more members we recruit to the tribe, the more successful we will be at addressing the problem of falls.

## Conflict of Interest Statement

The author declares that the research was conducted in the absence of any commercial or financial relationships that could be construed as a potential conflict of interest.

This paper is included in the Research Topic, “Evidence-Based Programming for Older Adults.” This Research Topic received partial funding from multiple government and private organizations/agencies; however, the views, findings, and conclusions in these articles are those of the authors and do not necessarily represent the official position of these organizations/agencies. All papers published in the Research Topic received peer review from members of the Frontiers in Public Health (Public Health Education and Promotion section) panel of Review Editors. Because this Research Topic represents work closely associated with a nationwide evidence-based movement in the US, many of the authors and/or Review Editors may have worked together previously in some fashion. Review Editors were purposively selected based on their expertise with evaluation and/or evidence-based programming for older adults. Review Editors were independent of named authors on any given article published in this volume.
